# The prevalence and demographic associations of headache in the adult population of Benin: a cross-sectional population-based study

**DOI:** 10.1186/s10194-024-01760-z

**Published:** 2024-04-05

**Authors:** Thierry Adoukonou, Mendinatou Agbetou, Eric Dettin, Oyene Kossi, Andreas Husøy, Hallie Thomas, Dismand Houinato, Timothy J Steiner

**Affiliations:** 1 Department of Neurology, University of Parakou, Parakou, Benin; 2 Department of Neuromedicine and Movement Science, NorHEAD, Norwegian University of Science and Technology (NTNU), Edvard Griegs gate, Trondheim, Norway; 3 Department of Neurology, University of Copenhagen, Copenhagen, Denmark; 4 Division of Brain Sciences, Imperial College London, London, UK

**Keywords:** Epidemiology, Prevalence, Population-based study, Headache, Migraine, Tension-type headache, Medication-overuse headache, Benin, Sub-Saharan Africa, Global Campaign against Headache

## Abstract

**Background:**

The Global Burden of Disease (GBD) study is increasingly well informed with regard to headache disorders, but sub-Saharan Africa (SSA) remains one of the large regions of the world with limited data directly derived from population-based studies. The Global Campaign against Headache has conducted three studies in this region: Ethiopia in the east, Zambia in the south and Cameroon in Central SSA. Here we report a similar study in Benin, the first from West SSA.

**Methods:**

We used the same methods and questionnaire, applying cluster-randomized sampling in three regions of the country, randomly selecting households in each region, visiting these unannounced and randomly selecting one adult member (aged 18–65 years) of each household. The HARDSHIP structured questionnaire, translated into Central African French, was administered face-to-face by trained interviewers. Demographic enquiry was followed by diagnostic questions based on ICHD-3 criteria.

**Results:**

From 2,550 households with eligible members, we recruited 2,400 participants (participating proportion 94.1%). Headache ever was reported by almost all (95.2%), this being the lifetime prevalence. Headache in the last year was reported by 74.9%. Age-, gender- and habitation-adjusted estimates of 1-year prevalence were 72.9% for all headache, 21.2% for migraine (including definite and probable), 43.1% for TTH (also including definite and probable), 4.5% for probable medication-overuse (pMOH) and 3.1% for other headache on ≥ 15 days/month. One-day (point) prevalence of headache was 14.8% according to reported headache on the day preceding interview.

**Conclusions:**

Overall, these findings are evidence that headache disorders are very common in Benin, a low-income country. The prevalence of pMOH, well above the estimated global mean of 1–2%, is evidence that poverty is not a bar to medication overuse. The findings are very much the same as those in a similar study in its near neighbour, Cameroon. With regard to migraine, they are reasonably in accord with two of three earlier studies in selected Beninese populations, which did not take account of probable migraine. This study adds to the hitherto limited knowledge of headache in SSA.

## Background

Headache disorders are increasingly recognized as a major cause of diminished health, impaired quality of life and lost productivity [1]. Primary headache disorders are highly prevalent: tension-type headache (TTH) affects an estimated 26.0% of people in the world, and migraine 14–15% [2, 3]. Furthermore, they most affect adults in their productive years [4]. Yet, while knowledge of headache prevalence and attributed burden worldwide has improved very substantially over the last two decades [3], in parts of the world these are still poorly described. Sub-Saharan Africa (SSA) in particular remains one of the large geographical areas with limited data derived from population-based studies.

The Global Campaign against Headache, led by the UK-registered *Lifting The Burden* (LTB) in official relations with the World Health Organization, has conducted three studies in this region: Ethiopia [5] in the east, Zambia [6] in the south and Cameroon [7] in Central SSA. Here we report a similar study in Benin, the first from West SSA.

Located on the coast of West Africa and bordered by Niger, Burkina Faso, Nigeria and Togo, Benin has a population estimated at 13.6 million, of whom approximately half live in urban areas [8]. It is among the world’s poorest countries, ranked by gross domestic product per purchasing power in 157th place [9]. Benin has one of the world’s highest death rates for children under five years, and, largely but not entirely because of this, a life expectancy of just 59 years; both are testimony to scarce access to a health-care system that is underdeveloped [8]. Nevertheless, because of a high fertility rate, Benin has a young population, with a median age of 17 years [8]. In other words, less than half its population are adult (aged ≥ 18 years).

In such circumstances, allocation of health resources to headache requires compelling evidence of need and expected benefit. However, published epidemiological data on headache in Benin are limited and conflicting. Of only three studies conducted in Benin, one, performed as a door-to-door survey in a localized semi-urban community in the north of the country, found a prevalence of TTH of 26.9% and of migraine of 14.4% [10], very close to global estimates [2]. Another, conducted a decade earlier in a rural community, but also performed as a door-to-door survey, found a migraine prevalence of only 3.3% [11]. A third study found 11.3% of university students to have migraine [12]. Methodological differences, especially between sampling procedures, probably explain the discrepant findings.

The aims of this study were to estimate the prevalence of the most common headache disorders (migraine, TTH and medication-overuse headache [MOH]), and explore their relationships with demographic and social status factors in the general population of Benin using LTB’s established expert-consensus-based methodology [13]. This study was undertaken as a project within the Global Campaign against Headache.

## Methods

### Ethics

The protocol was approved by the Local Ethics Committee for Biomedical Research of the University of Parakou (CLERB-UP) under number 0168/CLERB-UP/P/SP/SA of April 10, 2019. The study was conducted in accordance with the Declaration of Helsinki [14].

Necessary authorizations from academic and administrative authorities were obtained. All participants were informed of the nature and purpose of the study and gave oral consent before enrolment. Anonymity and confidentiality of the information collected were respected in accordance with data-protection laws.

### Study design

This was a cross-sectional study of the adult general population in Benin, using cluster-sampling to select a representative sample. In unannounced door-to-door visits to households, trained interviewers randomly selected, and interviewed, one member of each aged 18–65 years.

Pre-pilot and pilot studies tested the questionnaire and methods prior to the main study.

### Pre-pilot study

The pre-pilot study was carried out at the Centre Hospitalier Universitaire et Départemental over a period of 1 month using the first draft of the questionnaire to test its acceptability and comprehensibility. Respondents were 80 adults (aged 18–65 years), of whom 40 were patients presenting with headache and 40 were accompanying persons not complaining of headache. The study provided the basis for finalisation of the questionnaire.

### Questionnaire

Interviewers used the Headache-Attributed Restriction, Disability, Social Handicap and Impaired Participation (HARDSHIP) structured questionnaire developed by LTB [15], in the Central African French version used in Cameroon [7]. This questionnaire, described in detail previously, consists of several modules, three of which were used here: the demographic and social status module (enquiring into gender, age, habitation [urban or rural], marital status, education level and household income), and the headache diagnostic and description modules [15]. All participants completed the first module and answered the screening questions of the second (“have you ever had headache?” and “have you had headache during the last year?”). Only those responding positively to the latter question continued with the headache modules, describing their most bothersome headache if they had more than one type. Two questions asked whether headache had occurred on the day prior to the interview (“headache yesterday” [HY]) and whether, if so, it was of this type.

### Study areas

**Fig. 1:**
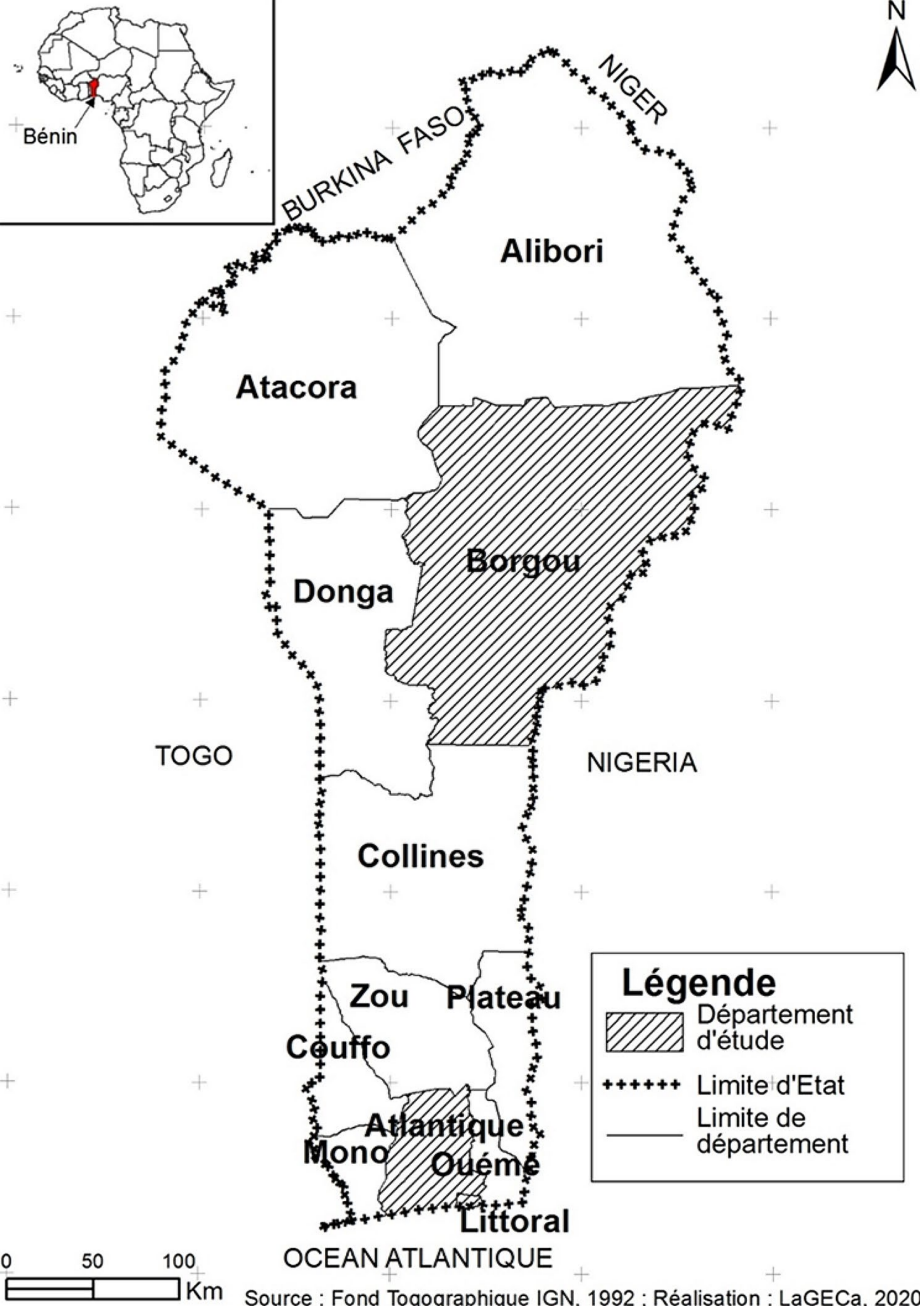
Map of administrative departments of Benin indicating those surveyed (hatched)

To obtain a representative sample of the general population of Benin, we recruited from three geographical regions (departments): Borgou (Parakou [urban] and Pèrèrè [rural]), Atlantique (Torri-Bossito [rural]) and Littoral (Cotonou [urban]) (Fig. 1).

### Interviewers

The 12 interviewers were physicians or senior medical or epidemiology students. In a two-day session at Unité de Recherche Clinique et Epidémiologique (URCE), Faculty of Medicine, University of Parakou, they received instruction in headache disorders (basic epidemiological and clinical aspects) and the study (design, purpose and practical aspects). Competence was ensured in supervised interviews.

These interviewers conducted both the pilot and the main studies.

### Pilot study

The pilot study field-tested the final version of the questionnaire in communities in Porto-Novo, a city 40 km from Cotonou, and in the surrounding rural areas. Using a mix of convenience and purposive sampling, 160 non-biologically related adults (aged 18–65 years) were interviewed, 40 urban and 120 rural.

Data from the pilot study were not included in the main analysis, but provided an estimate (with confidence interval [CI]) of the expected refusal rate.

### Main study

This was completed between May 11th and July 4th, 2020.

#### Sampling

We used simple random sampling to select four towns in the three departments: Cotonou (urban) in Littoral; Tori-Bossito (rural) in Atlantique; and Parakou (urban) and Pèrèrè (rural) in Borgou (Fig. 1). In these we followed a three-stage sampling procedure. At the first level, we randomly selected 28 villages and/or town districts in rural areas and 12 town districts in urban areas. At the second level, we selected 30 dwellings per village or town district in a randomly chosen direction from a central starting point. At the third level, one individual per household was randomly selected.

First visits to dwellings were unannounced (“cold-calling”). At each, the interviewer identified the number of non-biologically related families living there (families connected by a first- or second-degree relative [parent, sibling, child, uncle, aunt, nephew, niece or first cousin] were considered as one). Each family was regarded as a household. The wife or head of household was asked to list all adult family members (aged 18–65 years) living there, from which one person (the participant) was selected by lottery. Only this person was eligible: if he or she was not then present, an appointment was made for interview. Refusals were counted, but not replaced from the household.

#### Sample size

The sample size of *N* = 2,400 was estimated for an expected prevalence of 24.8%, a precision of 1.8% and an anticipated refusal rate of 10%. Methodological guidelines recommend a minimum sample size of *N* = 2,000 [13].

#### Enquiry

At interview, demographic enquiry was followed by headache screening questions (“Have you ever had headache?” and “Have you had headache in the last year?”), with the full interview covering headache characteristics (symptoms, including those contributing to diagnosis of headache type, and attributed burden [not reported here]) proceeding only when answers to both were positive.

#### Quality control and data entry

As data were collected in each region, they were checked for quality by two supervising neurologists. Additionally, the principal investigator reviewed completed questionnaires as the study proceeded.

All data were entered twice into Excel at URCE under the supervision of a senior epidemiologist, and discrepancies resolved. The original questionnaires were retained securely in the unit.

### Analysis

Gender was recorded as a binary variable (male or female). Age was recorded as a continuous variable but later categorized: 18–25, 26–35, 36–45, 46–55 or 56–65 years. Habitation was recorded as urban or rural, marital status as single, married or widowed/separated/divorced, education level as none, primary school, secondary school or college+, and household income in West African francs (XOF) as < 10,000, 10,000–20,000, 20,001–50,000 or > 50,000. In June 2020, USD 1 = XOF 583.

### Headache diagnoses

Headache diagnoses were made algorithmically during analysis, according to responses to the HARDSHIP diagnostic module, which applied modified ICHD-3 criteria [16]. Only one diagnosis was allowed in each participant. The algorithm first identified those reporting headache on ≥ 15 days/month (H15+). Participants also reporting acute medication use on ≥ 15 days/month were categorized as having probable MOH (pMOH), on the assumption that only simple analgesics were available to the vast majority in a low-income country. The others were categorized as “other H15+”, with no further attempt at diagnosis. Participants reporting headache on < 15 days/month were categorized, as stipulated by ICHD-based criteria [16], in hierarchical order: definite migraine, definite TTH, probable migraine and probable TTH. Any remaining cases were unclassified.

### Statistics

Mean age, gender and habitation distributions in the sample were compared, using chi-squared tests, with those of Benin’s population aged 18–65 years.

We estimated 1-year prevalences as proportions (%) with 95% confidence intervals (CIs), reporting observed and age-, gender- and habitation-adjusted values for each headache type. We combined definite and probable migraine and definite and probable TTH in these and all further analyses. We estimated point prevalence of headache, as a proportion (%), from reported HY, and calculated the predicted point prevalence from the observed 1-year prevalence and mean headache frequency in days/month.

We used bivariate and multivariate analyses (calculating odds ratios [ORs] and adjusted ORs [aORs] with 95% CIs) to identify associations, if any, between each headache type and the demographic and social status variables. Significance was set at *p* < 0.05.

We used Microsoft Excel version 16 to calculate adjusted prevalences, and IBM-Statistical Package for Social Sciences statistical software (SPSS) version 28 (SPSS Inc., Chicago, IL) for all other analyses.

## Results

### Sample description

From 2,550 households with eligible members, we recruited 2,400 participants (participating proportion 94.1%). Of these, 50% were from Borgou, 35% from Atlantique and 15% from Littoral. The gender distribution in the sample (51.3% male) reflected that of Benin (49.8% male; chi-squared = 2.0; *p* = 0.15). Mean age in the sample was 32.1 years, similar in both genders, and slightly younger than the mean age of Benin’s inhabitants aged 18–65 years (34.6 years). Rural habitants were overrepresented (70% in the sample; 52% in the country as a whole; chi-squared = 311.5; *p* < 0.001).

### Headache prevalence

Reported lifetime prevalence (headache ever) was 95.2%. Observed 1-year prevalence (headache reported in the last year) was 74.9%, slightly higher in females (77.6%) than males (72.3%). Table 1 summarizes the observed 1-year prevalences of all headache and each headache type by gender. Only 0.7% of headaches were unclassified.

**Table 1:** Observed 1-year prevalences overall and by gender

Headache type	Overall % [95% CI]	Male % [95% CI]	Female % [95% CI]
**All headache**	74.9 [73.1–76.6]	72.3 [69.7–74.8]	77.6 [75.1–80.0]
**Migraine** definite probable	23.5 [21.8–25.3] 10.5 [9.3–11.8] 13.0 [11.7–14.5]	22.1 [19.9–24.6] 10.1 [8.5–11.9] 12.1 [10.4–14.1]	24.9 [22.4–27.5] 10.9 [9.1–12.8] 14.0 [12.1–16.2]
**Tension-type headache** definite probable	43.6 [41.6–45.6] 33.9 [32.0-35.9] 9.7 [8.6–11.0]	44.3 [41.6–47.2] 34.2 [31.6–36.9] 10.2 [8.6–12.0]	42.9 [40.0-45.8] 33.6 [30.9–36.4] 9.2 [7.6–11.1]
**pMOH**	4.2 [3.5–5.1]	2.5 [1.7–3.6]	6.0 [4.7–7.5]
**Other H15+**	2.8 [2.2–3.6]	2.9 [2.1-4.0]	2.7 [1.9–3.8]

pMOH: probable medication-overuse headache; H15+: headache on ≥ 15 days/month

TTH was by far the most prevalent headache (43.6%), almost twice as common as migraine (23.5%). H15 + was reported by 7.0%, categorized as pMOH in 4.2% and as other H15 + in 2.8 (Table 1). Age-, gender- and habitation-adjusted estimates differed somewhat from observed (any headache 72.9% [95% CI: 71.1–74.6]; migraine 21.2% [19.7–23.0]; TTH 43.1% [41.1–45.1]; pMOH 4.5% [3.7–5.4]; other H15 + 3.1% [2.4–3.9]).

**Table 2:** Bivariate analyses of associations with demographics and social status variables

Variable	Migraine	TTH	pMOH	Other H15+
	Odds ratio [95% confidence interval]			
**Gender**				
male (*n* = 1,231)	reference	reference	reference	reference
female (*n* = 1,169)	1.2 [1.0-1.4] *p* = 0.12	0.9 [0.8–1.1] *p* = 0.46	2.5 [1.6–3.8] *p* < 0.001	0.9 [0.6–1.5] *p* = 0.78
**Age (years)**				
18–25 (*n* = 864)	reference	reference	reference	reference
26–35 (*n* = 786)	1.0 [0.8–1.2] *p* = 0.67	1.0 [0.8–1.2] *p* = 0.91	1.4 [0.8–2.5] *p* = 0.19	0.9 [0.5–1.6] *p* = 0.81
36–45 (*n* = 440)	0.9 [0.7–1.2] *p* = 0.59	1.2 [1.0-1.5] *p* = 0.12	1.7 [0.9–3.1] *p* = 0.10	0.5 [0.2–1.2] *p* = 0.11
46–55 (*n* = 190)	0.9 [0.6–1.3] *p* = 0.43	0.8 [0.6–1.1] *p* = 0.21	3.7 [1.9–6.9] *p* < 0.001	1.0 [0.4–2.5] *p* = 0.98
56–65 (*n* = 120)	0.9 [0.6–1.5] *p* = 0.82	0.9 [0.6–1.3] *p* = 0.59	2.5 [1.1–5.7] *p* = 0.03	1.3 [0.5–3.6] *p* = 0.55
**Habitation**				
urban (*n* = 720)	reference	reference	reference	reference
rural (*n* = 1,680)	1.9 [1.5–2.4] *p* < 0.001	0.7 [0.6–0.8] *p* < 0.001	1.1 [0.7–1.8] *p* = 0.61	0.7 [0.4–1.2] *p* = 0.22
**Marital status**				
single (*n* = 710)	reference	reference	reference	reference
married (*n* = 1,575)	1.2 [1.0-1.5] *p* = 0.10	0.8 [0.7-1.0] *p* = 0.04	2.0 [1.2–3.4] *p* = 0.01	0.7 [0.4–1.1] *p* = 0.14
widowed, separated or divorced (*n* = 115)	0.8 [0.5–1.3] *p* = 0.42	1.1 [0.8–1.7] *p* = 0.54	4.3 [2.0-9.5] *p* < 0.001	1.2 [0.5–3.3] *p* = 0.66
**Education level**				
none (*n* = 691)	2.1 [1.5-3.0] *p* < 0.001	0.5 [0.4–0.6] *p* < 0.001	1.6 [0.8–2.9] *p* = 0.15	1.1 [0.5–2.2] *p* = 0.85
primary (*n* = 473)	1.7 [1.2–2.4] *p* = 0.004	0.7 [0.5–0.9] *p* = 0.006	1.2 [0.6–2.3] *p* = 0.62	1.0 [0.4–2.1] 0.90
secondary (*n* = 882)	1.6 [1.2–2.3] *p* = 0.004	0.7 [0.5–0.8] *p* = 0.001	0.7 [0.3–1.3] *p* = 0.21	0.7 [0.3–1.5] 0.40
college+ (*n* = 354)	reference	reference	reference	reference
**Household income (XOF)**				
< 10,000 (*n* = 758)	1.4 [1.1–1.9] *p* = 0.01	0.9 [0.7–1.1] *p* = 0.21	1.1 [0.6–2.1] *p* = 0.73	4.8 [1.7–13.8] *p* = 0.003
10,000–20,000 (*n* = 575)	1.3 [1.0-1.7] *p* = 0.10	0.7 [0.5–0.9] *p* = 0.003	2.0 [1.1–3.7] 0.03	4.4 [1.5–12.8] *p* = 0.008
20,001–50,000 (*n* = 579)	1.0 [0.8–1.4] *p* = 0.75	0.8 [0.6-1.0] *p* = 0.06	1.5 [0.8–2.8] *p* = 0.23	3.2 [1.1–9.8] *p* = 0.04
> 50,000 (*n* = 488)	reference	reference	reference	reference

TTH: tension-type headache; pMOH: probable medication-overuse headache; H15+: headache on ≥ 15 days/month

**Table 3:** Multivariate analyses of associations with demographic and social status variables

Variable	Migraine	TTH	pMOH	Other H15+
	Adjusted odds ratio [95% confidence interval]			
**Gender**				
male	reference	reference	reference	reference
female	1.1 [0.9–1.3] *p* = 0.58	1.0 [0.8–1.2] *p* = 0.84	2.5 [1.6–4.1] *p* < 0.001	0.7 [0.4–1.3] *p* = 0.25
**Age (years)**				
18–25	reference	reference	reference	reference
26–35	1.0 [0.7–1.3] *p* = 0.89	1.0 [0.8–1.3] *p* = 0.79	1.3 [0.7–2.4] *p* = 0.35	1.2 [0.6–2.2] *p* = 0.65
36–45	1.0 [0.7–1.3] *p* = 0.92	1.3 [1.0-1.7] *p* = 0.07	1.5 [0.8–3.1] *p* = 0.22	0.7 [0.3–1.7] *p* = 0.39
46–55	1.0 [0.6–1.5] *p* = 0.89	0.8 [0.6–1.2] *p* = 0.32	3.5 [1.6–7.4] *p* = 0.001	1.3 [0.5–3.6] *p* = 0.65
56–65	1.0 [0.6–1.7] *p* = 0.93	0.9 [0.6–1.4] *p* = 0.73	2.2 [0.9–5.7] *p* = 0.10	1.5 [0.5-5.0] *p* = 0.47
**Habitation**				
urban	reference	reference	reference	reference
rural	1.7 [1.3–2.2] *p* < 0.001	0.8 [0.7-1.0] *p* = 0.04	1.2 [0.7–1.9] *p* = 0.57	0.6 [0.3-1.0] *p* = 0.07
**Marital status**				
single	reference	reference	reference	reference
married	1.1 [0.9–1.5] *p* = 0.30	0.9 [0.7–1.1] *p* = 0.32	1.2 [0.6–2.3] *p* = 0.60	0.9 [0.5–1.7] *p* = 0.72
widowed, separated or divorced	0.7 [0.4–1.3] *p* = 0.31	1.4 [0.9–2.2] *p* = 0.18	1.4 [0.5–3.6] *p* = 0.55	1.3 [0.4–4.7] *p* = 0.67
**Education level**				
none	1.4 [1.0-2.1] *p* = 0.08	0.6 [0.4–0.8] *p* < 0.001	0.8 [0.3–1.7] *p* = 0.49	1.0 [0.4–2.6] *p* = 0.95
primary	1.2 [0.8–1.8] *p* = 0.35	0.8 [0.6–1.1] *p* = 0.12	0.7 [0.3–1.7] *p* = 0.47	0.8 [0.3–2.1] *p* = 0.71
secondary	1.2 [0.9–1.8] *p* = 0.22	0.7 [0.6-1.0] *p* = 0.03	0.5 [0.2-1.0] *p* = 0.04	0.7 [0.3–1.6] *p* = 0.38
college+	reference	reference	reference	reference
**Household income (XOF)**				
< 10,000	1.2 [0.9–1.6] *p* = 0.29	1.0 [0.8–1.4] *p* = 0.82	1.0 [0.5–2.3] *p* = 0.89	6.1 [1.9–19.0] *p* = 0.002
10,000–20,000	1.0 [0.8–1.4] *p* = 0.77	0.8[0.6–1.1] *p* = 0.16	1.9 [0.9–3.9] *p* = 0.08	5.2 [1.7–16.4] *p* = 0.004
20,001–50,000	0.9 [0.7–1.3] *p* = 0.58	0.9 [0.7–1.1] *p* = 0.36	1.5 [0.7–2.9] *p* = 0.29	3.7 [1.2–11.7] *p* = 0.02
> 50,000	reference	reference	reference	reference

TTH: tension-type headache; pMOH: probable medication-overuse headache; H15+: headache on ≥ 15 days/month

Almost one in five (19.4%) of those with headache in the last year reported HY, equating to 14.8% [95% CI: 13.4–16.3] of the total sample, this being the point prevalence. The predicted point prevalence, from 1-year prevalence of any headache (74.9%) and mean headache frequency (4.3 days/month), was 10.7%. As expected, high proportions of those with pMOH (58.4%) and other H15+ (38.2%) reported HY. For migraine and TTH, the proportions were 18.1% and 11.0% respectively.

### Associations

Bivariate and multivariate association analyses are shown in Tables 2 and 3. Numbers for other H15 + were small in many of these analyses.

With regard to gender, the higher prevalence of migraine among females (Table 1) was not significant in either analysis. TTH was not associated with gender (aOR = 1.0). pMOH was strongly associated with female gender (aOR = 2.5; *p* < 0.001), but there was no similar association for other H15+.

Prevalence of migraine was highest among those aged 18–35 years (Table 2), but variations were small and not significant. pMOH became increasingly prevalent with advancing age (Table 2). Other H15 + showed no association with age.

Migraine was associated with rural dwelling, TTH with urban dwelling. pMOH was least prevalent among those who were single, and most prevalent among those who were widowed, separated or divorced (Table 2), but these lost significance in multivariate analysis (Table 3). Migraine was negatively associated with educational level (Table 2), although this appeared only as a non-significant trend in multivariate analysis (Table 3). TTH was positively associated with educational level in both analyses. Migraine was most prevalent among those with least income (Table 2), but again this lost significance in multivariate analysis (Table 3). Other H15 + showed a strong negative association with household income (aOR = 6.1 for those in the lowest category) (Table 3).

## Discussion

This study in Benin continued the series of adult population-based studies in SSA conducted within the Global Campaign against Headache. Its purposes were to add to knowledge of headache and to inform health policy in the region. It was the first such study in West SSA, following those in Central SSA (Cameroon [7]) and in the east (Ethiopia [5]) and south (Zambia [6]). Headache ever (observed lifetime prevalence) was reported by almost all participants (95.2%). Age-, gender- and habitation-adjusted estimates of 1-year prevalence were 72.9% for all headache, 21.2% for migraine (including definite and probable), 43.1% for TTH (also including definite and probable), 4.5% for pMOH and 3.1% for other H15+.

One-day (point) prevalence of headache was 14.8% according to reported HY, higher than the 10.7% predicted from 1-year prevalence and reported mean headache frequency. The former was presumably a more reliable estimate, not being subject to recall error.

Overall, these findings are evidence that headache disorders are very common in Benin, a low-income country. The prevalence of pMOH (4.5%), well above the estimated global mean of 1–2% [17–19], is of particular note: poverty, apparently, is not a total hindrance against overuse of acute treatment. We found no association between pMOH and household income in multivariate analysis.

Association analyses were notable for what they did not find. With an unusually high observed prevalence among males (22.1%), migraine was only weakly (and not significantly) associated with female gender, in contrast to almost universal findings elsewhere [2, 20]. Neither was migraine significantly associated with age. The sample was young (mean age 32.1 years: a reflection of Benin’s low life expectancy), which possibly influenced these findings. pMOH, on the other hand, showed clear associations with both female gender and advancing age, which were expected [21].

Migraine was associated with rural dwelling, and most prevalent among those with least education and least income (although these lost significance in multivariate analysis since both were also associated with rural dwelling). TTH was more common in the presumably more stressful urban areas, and least common among the least educated (retaining significance). Here a potential for interaction should be recognised, since diagnoses were mutually exclusive, with precedence given to migraine. This meant not only that any participant with both would be diagnosed only with migraine but also, in association analyses, each headache type was in the control group of the other (e.g., positive for migraine was compared with negative for migraine, the latter including those with TTH or other headache as well as the relatively few without headache).

In the unadjusted analysis, pMOH was least common among single people, which probably had less to do with marital status than with age (single people being generally younger), since it lost significance in multivariate analysis.

There are some other data from Benin for comparison. Migraine prevalence estimates in two previous studies, of 11.3% and 14.4%, probably included definite migraine only (they are not explicit on this) [10, 12]. The former found, as we did, TTH (presumably also definite only) to be twice as common as migraine [10]. These studies were in urban or semi-urban samples, and the latter was among university students only. Our estimate for definite migraine was 10.5%, not very dissimilar given the differences in sampling between the studies. An earlier rural study was discrepant, reporting a migraine prevalence of only 3.3% [12]. This study, well conducted, with door-to-door enquiry and an adequate sample size, used a diagnostic questionnaire based on ICHD-I [22], which seems the most likely reason for the difference.

More enlightening may be comparisons with the Global Campaign study in Cameroon [7], since it used the same methodology and questionnaire as this study in Benin. The two countries, both former French colonies, are not far distant in Central and West SSA respectively, although their tribal origins differ, and Benin is a poorer country. These comparisons, set out in Table 4, show similar estimates for the two countries, with very high prevalences of TTH compared with the global mean of 26.0% [2]. There was somewhat less migraine in Cameroon but significantly more pMOH and other H15+. As noted in the publication, since pMOH and migraine in these studies were mutually exclusive diagnoses, and migraine is the usual progenitor headache for MOH, the proportion diagnosed with migraine is depleted by cases proceeding to and diagnosed with pMOH. In both countries, pMOH was common, although Benin did not show the association of pMOH with urban dwelling that was very evident in Cameroon (and also in Zambia, in another similar study [6]). The threshold adopted for acute medication overuse in both Benin and Cameroon was ≥ 15 days/month, and in Zambia > 3 days/week, presuming, in each case, only simple analgesics to be available to the vast majority. Other H15 + was also common in both countries, but doubly so in Cameroon. We did not attempt to diagnose these further, since the necessary detailed characterization of headache is not possible in cross-sectional surveys with a single encounter, but we speculated that, in Cameroon, malaria might be a contributor [7]. Malaria is endemic in both countries, with headache a cardinal symptom, and this might explain the very high lifetime prevalence reported in both countries (95.2% in Benin, 94.8% in Cameroon [7]).

**Table 4:** One-year prevalence estimates for Benin and Cameroon, adjusted for age, gender and habitation, for each headache type

Headache type	Benin [this study]	Cameroon [7]
	Adjusted prevalence estimate % [95% confidence interval]	
**Migraine** (definite + probable)	21.2% [19.7–23.0]	18.1 [16.8–19.5]
**Tension-type headache** (definite + probable)	43.1% [41.1–45.1]	44.8 [43.1–46.6]
**Probable medication-overuse headache**	4.5% [3.7–5.4]	6.5 [5.7–7.4]
**Other headache on ≥ 15 days/month**	3.1% [2.4–3.9]	6.6 [5.8–7.6]

### Study strengths and limitations

Strengths were the use of standardized, expert-consensus-based and well tested methods [13] and questionnaire [15], an adequate sample (*N* = 2,400) drawn from multiple regions of the country in order to be representative of its diverse population, and a low non-participating proportion (5.9%). While we oversampled in rural areas (where migraine was more prevalent), statistical correction of prevalence estimates took full account of this.

The standardized methodology is designed to reduce the inherent limitations of questionnaire-based surveys. However, we were not able to test the validity of the diagnostic questions in the Central African French translation, since the resources for the necessary study (re-diagnosis of a subsample by a headache specialist) were not available in Benin. We relied on previous use of the same translation in Cameroon [7], and of the questionnaire in earlier Global Campaign studies in 19 languages [23], with validation studies in six [24–29]. In a cross-sectional survey with single enquiry, it is not realistically possible to diagnose more than one headache type. The focus on the most bothersome type was necessary to maintain clarity of description. In participants with both migraine and TTH, it would have been the latter that was not identified, meaning TTH prevalence was underestimated. MOH could be diagnosed only on the basis of association of H15 + and reported medication overuse, without evidence of causation (hence probable). The questionnaire, for reasons stated earlier, could not further diagnose other H15+, which might include secondary headaches, but among the other diagnostic possibilities were chronic migraine and chronic TTH. These would have been lost from our prevalence estimates, but the overall prevalence of other H15 + was only 3.1%. The proportion of unclassified cases was very low (0.7%).

## Conclusion

Headache disorders are highly prevalent in Benin, with three quarters of the adult population reporting headache in the last year and more than one person in seven having headache on any particular day. This picture is very similar to that of its near neighbour, Cameroon. The high prevalence of pMOH (triple the global mean) is evidence that poverty does not preclude overuse of acute medication. This study adds to the hitherto limited knowledge of headache in SSA.

## Data Availability

The original data are held at Department of Neurology, University of Parakou, Benin, and the analytical set at Norwegian University of Science and Technology, Trondheim, Norway. When analyses are completed, anonymised data will be available on request for academic purposes, in line with the policy of the Global Campaign against Headache.

## Abbreviations

aOR: adjusted odds ratio
CI: confidence interval
d/m: days/month
GBD: Global Burden of Disease
HARDSHIP: Headache-Attributed Restriction, Disability, Social Handicap and Impaired Participation questionnaire
HY: headache yesterday
ICHD: International Classification of Headache Disorders
LTB: *Lifting The Burden*
MOH: medication-overuse headache
OR: odds ratio
pMOH: probable MOH
SD: standard deviation
SSA: sub-Saharan Africa
TTH: tension-type headache
URCE: Unité de Recherche Clinique et Epidémiologique (Faculty of Medicine, University of Parakou)
USD: United States dollar
WHO: World Health Organization
XOF: West African franc
